# Determinants of iron deficiency and anemia among Nunavimmiut: results from the *Qanuilirpitaa?* 2017 Nunavik Health Survey

**DOI:** 10.17269/s41997-023-00775-4

**Published:** 2023-05-10

**Authors:** Audrey Lavoie, Mélanie Lemire, Benoit Lévesque, Pierre Ayotte

**Affiliations:** 1 Département de médecine sociale et préventive, Université Laval, Quebec City, QC Canada; 2 Institut de biologie intégrative et des systèmes, Université Laval, Quebec City, QC Canada; 3 Axe santé des populations et pratiques optimales en santé, Centre de recherche du CHU de Québec-Université Laval, Quebec City, QC Canada; 4 Institut national de santé publique du Québec, Quebec City, QC Canada

**Keywords:** Anaemia, Iron deficiency, Haemoglobin, Ferritin, Inuit, Nunavik, Anémie, carence en fer, hémoglobine, ferritine, Inuit, Nunavik

## Abstract

**Objective:**

To estimate the prevalence of iron deficiency (ID) and anemia and study their main distal and proximal protective and risk factors among Nunavimmiut 16 years and older in 2017.

**Methods:**

In a cross-sectional participatory survey of 831 women and 436 men from the *Qanuilirpitaa?* 2017 Nunavik Inuit Health Survey, venous blood samples were collected to measure various indicators of iron status and anemia as well as biomarkers of nutritional and inflammatory status and contaminant exposures. Sociodemographic, food security status, anthropometric, lifestyle, dietary, and health data were collected using questionnaires, clinical sessions, and a medical chart review. ID and anemia diagnoses were based on serum ferritin (SF) and hemoglobin (Hb), respectively. Multiple regressions were used to assess correlates of anemia and iron status.

**Results:**

Prevalence of ID was highest among women of childbearing age (16–49 years old, 33%) and anemia among adults aged 50 years and older (31%). These estimates are prone to biases due to the relatively low participation rate (37%). Serum vitamin D, omega-3 polyunsaturated fatty acid content of erythrocyte membranes, blood selenium, inflammation, higher socioeconomic status (SES), obesity, and alcohol consumption were all positively associated with SF, while *Helicobacter pylori* infection and a recent pregnancy were negatively associated with Hb among women of childbearing age. Among older adults, food insecurity was associated with lower SF.

**Conclusion:**

While data reported here provide some indication of an improvement since the previous survey conducted in 2004, additional efforts should be devoted to further increasing the SES and access to country foods and nutritious market foods in this population, the two main protective factors against ID and anemia identified in the present study.

**Supplementary Information:**

The online version contains supplementary material available at 10.17269/s41997-023-00775-4.

## Résumé

**Objectif:**

Estimer la prévalence de la carence en fer (CF) et de l’anémie et étudier leurs principaux facteurs de protection et de risque distaux et proximaux chez les Nunavimmiut de 16 ans et plus en 2017.

**Méthodologie:**

Dans le cadre de l’enquête transversale participative *Qanuilirpitaa?* menée en 2017 auprès de 831 femmes et 436 hommes, des échantillons de sang furent prélevés pour mesurer divers indicateurs de la CF et de l’anémie ainsi que des biomarqueurs de l’état nutritionnel et inflammatoire et de l’exposition aux contaminants environnementaux. Des données sociodémographiques, anthropométriques, sur la sécurité alimentaire, sur le mode de vie, l’alimentation et la santé ont été recueillies à l’aide de questionnaires, de séances cliniques et d’un examen des dossiers médicaux. Les diagnostics de CF et d’anémie furent basés sur la ferritine sérique (SF) et l’hémoglobine (Hb), respectivement. Des régressions multiples ont été utilisées pour évaluer les déterminants de l’anémie et du statut en fer.

**Résultats:**

La prévalence de la CF était la plus élevée chez les femmes en âge de procréer (16 à 49 ans, 33 %) et l’anémie chez les adultes âgés de 50 ans et plus (31 %). Ces estimés pourraient être biaisés puisque le taux de participation à l’enquête était relativement faible (37 %). Chez les femmes en âge de procréer, la vitamine D sérique, la teneur en acides gras polyinsaturés oméga-3 des membranes érythrocytaires, le sélénium sanguin, l’inflammation, un statut socio-économique plus élevé, l’obésité et la consommation d’alcool étaient tous associés positivement à la SF, tandis que l’infection à *Helicobacter pylori* et une grossesse récente étaient associées négativement à l’Hb. Chez les adultes plus âgés, l’insécurité alimentaire était associée à une diminution de la SF.

**Conclusion:**

Bien que les données de cette enquête suggèrent une amélioration depuis l’enquête précédente réalisée en 2004, des efforts additionnels sont requis pour améliorer le statut socioéconomique et l’accès aux aliments traditionnels et aux aliments de marché de qualité au Nunavik, les deux principaux facteurs protecteurs de la CF et de l’anémie identifiés dans la présente étude.

## Introduction

Anemia is a condition in which blood hemoglobin (Hb) concentration is insufficient to meet physiological needs. It affects about a third of the world’s population (World Health Organization (WHO), 2017). Anemia can lead to decreased work productivity, impaired cognitive and behavioural development, increased hospitalization, morbidity, and mortality (Beard, 2001).

Nunavik, one of the four regions of the Inuit Nunangat, is located north of the 55^th^ parallel in the province of Quebec, Canada. Approximately 11,000 Nunavimmiut (Inuit living in Nunavik) live in 14 communities scattered along the coast of Hudson Bay, Hudson Strait, and Ungava Bay. Although in the general Canadian population, the prevalence of anemia—around 3%—is one of the lowest in the world (Cooper et al., 2012), this is not the case in the Canadian Arctic. The prevalence of anemia documented among women in the framework of the *Qanuippitaa?* 2004 Nunavik Inuit Health Survey was 43% (Plante et al., 2011), compared to approximately 4% among women from the general Canadian population in 2009–2011 (Cooper et al., 2012). The elevated prevalence of anemia among Nunavik women represented a severe public health problem based on the WHO severity criteria (McLean et al., 2009). Nunavimmiut have been experiencing an important dietary transition in the past decades, characterized by a decrease of nutrient-rich country foods (many of which are rich in heme iron) and an increase in nutrient-poor market foods (Blanchet & Rochette, 2008).

The etiology of anemia is complex and multifactorial. In the context of populational studies, there are three main types of anemia: iron deficiency anemia (IDA), anemia of chronic inflammation (ACI), and unexplained anemia (UA). UA comprises anemia etiologies that are difficult to identify in such studies (Jamieson, 2012; Plante et al., 2011).

Iron deficiency (ID) is the primary cause of anemia, accounting for nearly half the cases worldwide (WHO, 2017). ID progresses through three overlapping stages: iron depletion, iron-deficient erythropoiesis, and IDA. Consequences of ID arise as early as during the first iron depletion stage in the form of fatigue, decreased energy, and impaired immune system functioning (Beard, 2001). In populational studies, iron depletion and iron-deficient erythropoiesis have been combined into iron deficiency without anemia (IDWA) due to the difficulty of a mutually exclusive diagnosis (Jamieson et al., 2016; Plante et al., 2007).

**Fig. 1:**
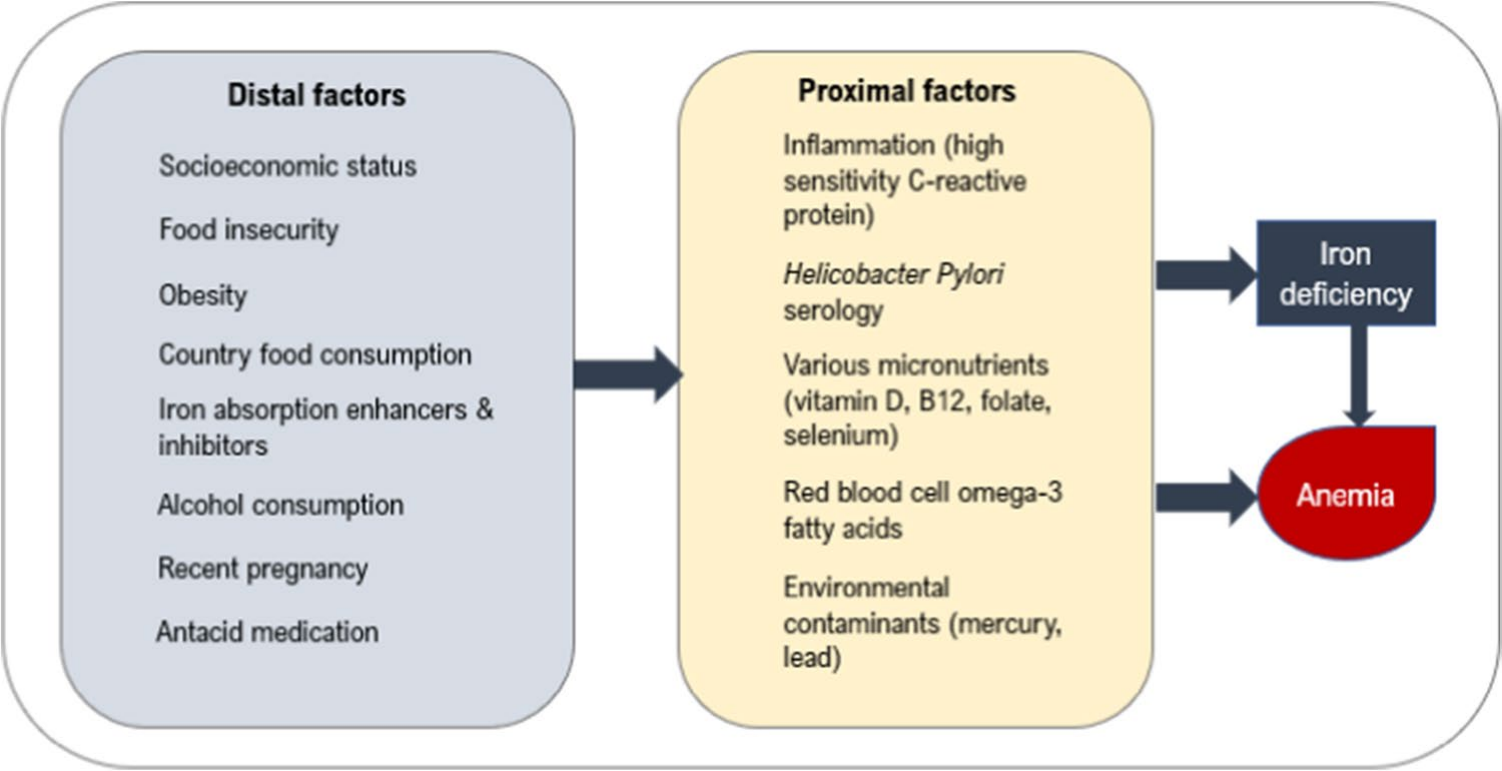
Conceptual causal chain of distal and proximal factors leading to iron deficiency and anemia

ID and anemia are influenced directly by proximal factors (e.g. nutrient deficiencies, inflammation, and exposure to environmental contaminants) that are assessed using biological biomarkers (Barabino, 2002; Fishman et al., 2000; Jamieson et al., 2013; Nemeth & Ganz, 2006; Semba et al., 2006). In addition, several distal factors (e.g. socioeconomic factors, dietary and lifestyle habits) are causally involved in ID and anemia (Fig. 1) (Aigner et al., 2014; Harrison-Findik et al., 2006; Hurrell & Egli, 2010; Pirkle et al., 2014). These factors are further explained in Supplementary Materials.

The previous health survey conducted in Nunavik (*Qanuippitaa?* 2004) documented the prevalence of anemia and ID in women only (Plante et al., 2011). Up to now, no study has realized an estimation of ID and anemia prevalence and a detailed assessment of their risk and protective factors using multivariate analysis in the whole Nunavik population aged 16 years and older. When a health outcome is explained through different levels of risk factors (distal and proximal), the use of different statistical models for each level is recommended to better explain the outcome (WHO, 2002). Additionally, knowledge of distal factors for any health outcome provides insight for implementing public health primary prevention interventions (Ancelle, 2017).

The objectives of the present study were to (a) estimate the prevalence of IDWA, IDA, and other types of anemia among Nunavimmiut 16 years and older according to sex and age groups; (b) identify distal and proximal factors modulating Hb and SF levels according to sex and age groups; and (c) identify distal and proximal protective and risk factors of IDWA and IDA among women of childbearing age (16 to 49 years old).

## Methods

### Community engagement and ethics

The *Qanuilirpitaa?* 2017 Nunavik Inuit Health Survey was set up following a resolution adopted by the Nunavik Regional Board of Health and Social Services (NRBHSS) requesting that a new health survey be conducted to update the information on the health status of Nunavimmiut. This survey was conducted in partnership with major Nunavik organizations, the Institut national de santé publique du Québec, and researchers from Université Laval, McGill University, and Trent University. An Inuit-led steering committee oversaw the preparation, conduct, data interpretation, and dissemination of the survey results. A data management committee (DMC) evaluated the usefulness of the research questions for the region, and approved data and biological sample requests. This committee brings together representatives from the NRBHSS and the health centres, the Kativik Regional Government, Makivik Corporation, Kativik Ilisarniliriniq, Avataq Cultural Institute, and Qarjuit Youth Council. The DMC met with the researchers to discuss results and provide co-interpretation of the data that takes into consideration Inuit culture and values, according to a “two-eyed seeing approach”. Comments provided by DMC members were considered in preparing the final version of the manuscript, which was approved by the DMC. Findings were communicated to the population and partners through infographics and summaries (in Inuktitut, English, and French), reports (in English), and live presentations. The *Qanuilirpitaa*? 2017 survey was also approved by the Comité d’éthique de la recherche du CHU de Québec – Université Laval. Informed written consent was obtained from each participant, and a clinical follow-up for abnormal results was undertaken when needed.

### Study population and design

Data collection took place in the 14 communities of Nunavik from August 19 to October 5, 2017. The targeted population was permanent residents of Nunavik aged 16 and older. The health survey methodology was detailed elsewhere (Hamel et al., 2020). Briefly, proportional sampling stratified by sex, age group, and community was used to select participants from the Makivik Corporation’s beneficiary list of Inuit living in Nunavik and noninstitutionalized. The participation rate was 79.7% among contacted individuals, but many sampled individuals could not be contacted (because they were out of the community) or missed their appointment due to bad weather or delays in the appointment schedule. Hence, the total response rate was 36.5%, with 31% for those aged 16–30 years and 42% for those aged 30 years and older. Participants were invited on board the Canadian Coast Guard Ship (CCGS) Amundsen to complete questionnaires and attend clinical sessions during which biological samples and anthropometric measurements were obtained. Blood samples were collected from 1325 participants. Following the exclusion of non-Inuit residents (*n* = 25) and pregnant women (*n* = 33), the final study sample consisted of 1267 participants.

### Biological samples and laboratory analyses

Blood samples were collected by research nurses. Whole blood Hb analysis was performed within 90 min after collection by a hematologist technician using the portable DxH500 hematology analyzer from Beckman-Coulter (Pasadena, CA, USA). Hb liberated following red blood cell (RBC) hemolysis is quantified by the cyanmethemoglobin method (Whitehead et al., 2019).

Clinical biochemistry analyses were performed at the Institut universitaire de cardiologie et de pneumologie de Québec (IUCPQ, Québec, QC, Canada). Serum iron (SI), ferritin (SF), total iron binding capacity (TIBC), transferrin saturation (TSAT), vitamin D, vitamin B_12_, and erythrocyte folate were determined using the MODULAR ANALYTICS e170 from Roche Diagnostics GmbH (Mannheim, Germany). Serum high-sensitivity C-reactive protein (hs-CRP) was determined using the Integra 800 from Roche Diagnostics. The presence of inflammation was defined as a hs-CRP level ≥ 3 mg/L (Kushner & Antonelli, 2015).* Helicobacter pylori* antibody (IgG) analysis was carried out at the Hôpital de Chicoutimi (Saguenay, QC) using the Captia™ ELISA kit from Trinity Biotech (Bray, Ireland).

Whole blood total mercury (Hg), lead (Pb), and selenium (Se) concentrations were measured at the Centre de toxicologie du Québec (Quebec City, QC, Canada) using inductively coupled plasma mass spectrometry with the NexION® instrument from PerkinElmer (Waltham, MA, USA). Performance data for the method (M-592) are provided in Table S1 of Supplementary Materials.

RBC fatty acid composition was analyzed at the Laboratory of Nutritional Lipidomics of the University of Waterloo (ON, Canada) using the Varian 3900 gas chromatograph equipped with a DB-FFAP 15 m × 0.10 mm i.d. × 0.10 µm column and a flame ionization detector (Agilent, Mississauga, ON). Total RBC eicosapentaenoic acid (EPA), docosahexaenoic acid (DHA), and docosapentaenoic acid (DPA) were expressed as percent of total fatty acids (by weight) and will hereafter be referred to as RBC n-3 LC-PUFA.

Participants were also asked to provide a spot urine sample on board the CCGS Amundsen. Urinary cotinine concentration, a biomarker of cigarette smoke exposure, was determined at the IUCPQ laboratory using the EnSpire 2300 Multilabel Reader from PerkinElmer.

### ID and anemia categorization

We defined anemia as a Hb concentration below 130 g/L for men and below 120 g/L for women (WHO, 2011). Hb cut-off values were adjusted based on smoking status (+ 3 g/L for 10 to 19 cigarettes/day, + 5 g/L for 20 to 39 cigarettes/day, + 7 g/L for 40 cigarettes/day or more) (CDC, 1989; WHO, 2017).

ID was defined using a multiple-index model (Plante et al., 2011). In the absence of clinical inflammation (hs-CRP < 10 mg/L), ID was defined as SF < 15 µg/L. In the presence of clinical inflammation (hs-CRP ≥ 10 mg/L), ID was defined as SF < 50 µg/L. Low iron stores (SF = 15–20 µg/L), with at least two abnormal values among three iron status indicators (SI < 10 µmol/L, TSAT < 15% and/or TIBC ≥ 68 µmol/L) was also defined as ID (Greig et al., 2013; Vieira et al., 2007). IDA was defined as the presence of ID and anemia simultaneously, whereas IDWA was defined as the presence of ID without anemia.

### Clinical assessments, questionnaires, and medical chart reviews

Waist circumference was measured twice in centimetres by nurses or trained interviewers (Hamel et al., 2020). A third measurement was taken for participants whose waist measurements differed by more than 1 cm. The mean of the two concordant measurements was used in this analysis. Obesity was defined as a waist circumference > 102 cm for men and > 88 cm for women (Lau et al., 2007).

Questionnaires were administered to obtain information on health, food insecurity, socioeconomic status (SES), demographics, and dietary habits. Information on individual income and education was obtained in the sociodemographic questionnaire, and a socioeconomic index (0–1) was created using the mean of those two indicators. Details regarding construction of the socioeconomic index are provided in Supplementary Materials. Consumption frequencies of various food items in the last 3 months were assessed using a food frequency questionnaire (FFQ), on a scale of 1 to 7, 1 being “never or less than once a month” and 7 being “4 times a day or more”. Due to time constraints, it was decided to focus on the past 3-month period for the FFQ. Answers may be more valid if limited to the most recent period prior to the survey. The frequency of consumption of country foods rich in iron was defined as the mean of individual FFQ scores for 17 different food items: beluga *nikku* (dried meat) and meat, seal meat and liver, walrus meat, caribou *nikku* and meat, polar bear, muskox, ptarmigan or partridge, goose, fish (Arctic char, lake, brook and sea trout, salmon, and other fish), fish *pitsik* (dried fish), and mollusks. Market foods were also assessed but were not added to the final models due to lack of association with hemoglobin and ferritin. Hot beverage consumption was defined as the mean of individual FFQ scores for coffee, traditional teas, and teas with caffeine. Food insecurity was dichotomized as food secure or food insecure, based on an adapted version of the Household Food Security Survey Module (HFSSM). Questions in the HFSSM were modified to be asked at the individual scale, to include both market and country foods and to refer in all questions not only to “money to buy food”, as in the original tool, but to “resources to get food”, in order to cover other means through which Nunavimmiut access food (Furgal et al., 2021). Daily alcohol consumption was quantified based on participants’ consumption frequency in the last 12 months and the number of drinks usually consumed on one occasion and was divided into 2 categories: less than 1 drink per day and 1 or more drinks per day.

A medical chart review was conducted by research nurses to obtain information on prescribed medication, specifically the use of any antacid medication.

### Statistical analysis

All statistical analyses were performed in SAS® Studio, Version 3.8 (Cary, NC, USA). Statistical weights were used to consider sampling methodology and item non-response. The detailed weighting process is presented in a separate methodology report (Hamel et al., 2020). Briefly, weights were attributed to participants based on age, sex, and community of residence. Additional weights were attributed based on survey item non-response. For all statistical analyses, a significance level of *α* < 0.05 was used (*α* < 0.10 for statistical trends), and variance was estimated using the balanced repeated replication (BRR) method.

Participants were divided by sex and age groups (16 to 49; 50 and older). Descriptive statistics were used to provide an overview of participant characteristics and prevalence of IDWA, IDA, and other types of anemia (OA). OA was defined by the presence of anemia without ID, essentially combining ACI and UA, whose prevalence was reported elsewhere (Lavoie et al., 2020). Three variables, Hg, Pb, and SF, were not normally distributed and were log transformed. Their geometric means are presented.

The percentage of missing data across all variables varied from 0% to 25% (Table S2, Supplementary Materials). Data were missing in a non-monotone multivariate pattern, and we used multiple imputation to create and analyze 50 imputed datasets (Rubin, 1987). Incomplete variables were imputed under fully conditional specification. All variables in the final analytical model were included in the imputation models, including dependent variables. Imputed observations for dependent variables were removed prior to data analysis. Categorical variables, log transformations, and interaction terms were created prior to the imputation and included in the imputation models.

Multiple linear regression analyses with imputed datasets were used to identify factors associated with Hb and SF among Nunavimmiut by sex and age groups. For each dependent variable, two models were created, one for proximal factors and one for distal factors. Multiple logistic regression was also used to study factors associated with IDWA and IDA only among women 16 to 49 years old, as these outcomes were not sufficiently prevalent to allow statistical analyses in other groups. The prevalence of OA was too low to perform multiple regression in any group.

Independent variables included in the models were selected according to a priori knowledge of their potential association with the outcome. All variables were kept in the model in order to better compare models between sex and age groups. This model-building strategy is consistent with published recommendations for explicative models in epidemiology (Ancelle, 2017). Variance inflation factors and tolerance were verified to rule out multicollinearity. An interaction term was tested between blood Se and Hg variables as several studies report a biological interaction between these two trace elements (Yang et al., 2008). Once analyses were completed on each imputed dataset, results were pooled according to Rubin’s rules.

## Results

### Study population characteristics

Descriptive characteristics of study participants by sex and age group are presented in Table 1. Mean Hb concentrations were lower and anemia prevalence was higher among older men and women compared to their younger counterparts. Overall, men exhibited higher Hb levels than women, while young men had a nearly 50% lower prevalence of anemia compared to women of reproductive age. SF concentration was lowest, and the prevalence of ID highest among women of childbearing age (16 to 49 years old).

**Table 1:** Characteristics of study participants

Variable	Women		Men	
	16 to 49 years old	50 years and older	16 to 49 years old	50 years and older
Age (years)	29.9 (29.4–30.4)^a^	58.7 (57.7–59.7)^b^	29.6 (28.9–30.3)^a^	60.0 (58.7–61.3)^b^
Blood hemoglobin (g/L)	129.2 (128.3–130.1)^b^	126.9 (125.9–128.7)^a^	144.8 (143.5–146.1)^d^	136.9 (134.4–139.4)^c^
Serum ferritin (µg/L)^1^	27.2 (25.2–29.2)^a^	58.9 (52.8–65.7)^b^	52.1 (47.1–57.7)^b^	64.0 (53.6–76.4)^b^
Anemia (%)				
Yes	20.3 (16.8–23.9)^b^	30.6 (24.1–37.2)^c^	11.4 (7.3–15.4)^a^	30.5 (21.8–39.2)^bc^
Iron deficiency (%)				
Yes	32.6 (28.7–36.6)^b^	7.2 (3.4–11.0)^a^	10.4 (6.6–14.3)^a^	9.6 (3.7–15.5)^a^
Proximal factors				
Serum vitamin D (nmol/L)	61.5 (59.4–63.6)^a^	105.9 (100.0–111.9)^b^	63.2 (60.3–66.0)^a^	97.9 (91.1–104.8)^b^
Serum vitamin B_12_ (pmol/L)	409.7 (399.6–419.7)^b^	469.7 (445.7–493.7)^c^	377.2 (362.9–391.5)^a^	420.1 (389.6–450.6)^ab^
RBC folate (nmol/L)	719.6 (708.8–730.5)^a^	732.6 (712.1–753.0)^a^	784.1 (766.4–801.8)^b^	787.9 (753.0–822.8)^b^
Blood total mercury (nmol/L)^1^	43.2 (40.2–46.5)^b^	75.8 (68.1–84.3)^c^	32.1 (28.0–36.7)^a^	64.3 (53.7–76.8)^c^
Blood total lead (µmol/L)^1^	0.10 (0.09–0.10)^a^	0.19 (0.18–0.21)^c^	0.12 (0.11–0.13)^b^	0.19 (0.17–0.22)^c^
Blood total selenium (µmol/L)	4.7 (4.4–4.9)^b^	5.5 (5.0–5.9)^c^	3.7 (3.4–4.0)^a^	4.9 (4.2–5.7)^bc^
RBC n-3 LC-PUFA (% of total fatty acids)^2^	7.4 (7.3–7.6)^b^	9.5 (9.2–9.9)^c^	6.5 (6.3–6.8)^a^	9.2 (8.7–9.8)^c^
Urinary cotinine (ng/mL)	1180 (1120–1240)^b^	899 (800–997)^a^	1150 (1063–1236)^b^	767 (643–891)^a^
hs-CRP (%)				
≥ 3 mg/L	24.7 (21.0–28.3)^ab^	29.7 (23.0–36.3)^b^	18.7 (13.8–23.5)^a^	30.0 (21.6–38.4)^b^
*H. pylori* serology (%)				
Positive	73.8 (70.2–77.4)^c^	51.7 (44.4–58.9)^a^	82.9 (78.6–87.2)^d^	63.8 (55.3–72.2)^b^
Distal factors				
Obesity (%)				
WC > cut-off	61.9 (57.8–65.9)^c^	63.0 (56.0–70.1)^c^	20.1 (15.2–25.1)^a^	40.7 (31.0–50.3)^b^
Food insecurity (%)				
Yes	77.8 (74.2–81.4)^b^	69.2 (61.8–76.5)^a^	82.5 (77.7–87.3)^b^	75.6 (67.8–83.4)^ab^
Socioeconomic index (0–1)	0.38 (0.36–0.40)	0.38 (0.34–0.42)	0.35 (0.33–0.37)	0.36 (0.32–0.40)
Country food rich in iron, frequency index (0–7)^3^	1.66 (1.63–1.69)^a^	1.73 (1.66–1.80)^ab^	1.84 (1.78–1.90)^b^	1.76 (1.68–1.84)^ab^
Hot beverages, frequency index (0–7)^3^	2.44 (2.38–2.50)^a^	3.10 (2.98–3.21)^c^	2.73 (2.63–2.84)^b^	3.29 (3.13–3.45)^c^
Alcohol consumption (%)				
≥ 1 drinks/day	44.9 (40.5–49.3)	39.0 (29.8–48.1)	38.2 (32.3–44.0)	34.3 (23.8–44.9)
Antacid medication (%)				
Yes	1.7 (0.7–2.8)^a^	9.0 (5.5–12.4)^b^	1.0 (0.1–1.9)^a^	11.8 (6.2–17.4)^b^

Arithmetic mean and percentage (95% CI). WC = waist circumference; WC cut-off: M > 102 cm, W > 88 cm. a, b, c, d, estimates with different letters are statistically different between groups based on *t* test with Tukey–Kramer correction for continuous variables and *χ*^2^ test for categorical variables

^1^Geometric mean presented

^2^Sum of EPA, DHA, and DPA expressed as % of total fatty acids

^3^Food frequency index based on FFQ

Serum vitamin D, blood Hg, Pb, Se, and RBC n-3 LC-PUFA concentrations were higher among older adults compared to the younger groups. Blood Hg, Se, and RBC n-3 LC-PUFA concentrations were also higher among women of childbearing age compared with younger men. Mean RBC folate levels were higher in men than in women, whereas the opposite was observed for serum vitamin B_12_ concentrations. *H. pylori* seroprevalence in younger adults was higher than among older groups and was most prevalent among younger men.

Obesity was more prevalent in women than in men. Country food consumption was lowest among women of childbearing age, and hot beverage consumption was highest among older adults.

Over 99% of Nunavimmiut met or exceeded the cut-off levels in the general population for deficiency of serum vitamin B_12_ and erythrocyte folate (data not shown). Additionally, very few had whole blood Pb concentrations over the Health Canada guidance value (3.3%; ≥ 0.5 µmol/L). These proportions were very low and did not permit further statistical analyses. More detailed results of descriptive and bivariate analyses were published elsewhere (Lavoie et al., 2020).

### Prevalence of IDWA, IDA, and other types of anemia

**Fig. 2:**
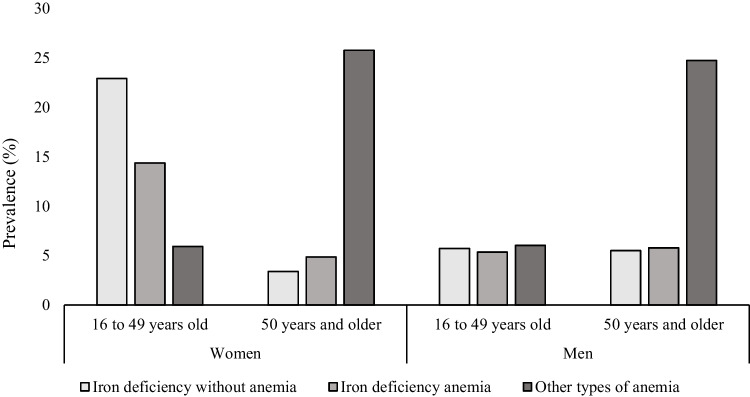
Prevalence of iron deficiency without anemia (IDWA), iron deficiency anemia (IDA), and other types of anemia (OA) among men and women, Nunavik, 2017

Among women of childbearing age, most cases of anemia (total prevalence = 20%) were IDA (14%), and 23% had IDWA. In contrast, among older men and women, most cases of anemia (total prevalence = 31% in each subgroup) were unrelated to ID (25% and 26%, respectively). Younger men displayed the lowest prevalence of anemia (11%), and less than half of cases (5%) were related to ID (Fig. 2).

### Proximal and distal factors associated with hemoglobin and ferritin status

Multiple linear regressions of Hb and SF as the dependent variables by sex and age groups are presented in Tables 2, 4, 5, and 6. Multiple logistic regressions of IDWA and IDA as dependent variables are presented in Table 3 for women of childbearing age. SF was positively associated with Hb concentrations in all subgroups, after adjusting for other factors. Other significant factors were identified in different sex and age subgroups.

**Table 2:** Multiple linear regression analyses of serum ferritin and blood hemoglobin concentrations among women of childbearing age (16 to 49 years old), Nunavik, 2017

	Ferritin (µg/L)^1^ (*n* = 602)		Hemoglobin (g/L) (*n* = 602)	
	Std*** β***	95% CI	***Std β***	95% CI
Proximal factors				
Serum ferritin (µg/L)^1^			.52**	.43 to .60
Age (years)	.02	− .07 to .10	.01	− .06 to .08
Urinary cotinine (ng/mL)	.05	− .03 to .13	.15**	.09 to .22
Serum vitamin D (nmol/L)	.12*	.01 to .22	− .01	− .08 to .07
Blood mercury (nmol/L)^1^	.06	− .06 to .18	.03	− .07 to .13
Blood selenium (µmol/L)	.17**	.07 to .27	.05	− .02 to .13
hs-CRP, (1 = ≥ 3 mg/L, 0 = < 3 mg/L)	.42**	.25 to .58	− .30**	− .47 to − .14
RBC n-3 LC-PUFA (% of total fatty acids)^2^	.13*	.01 to .26	− .02	− .13 to .08
*H. pylori* serology, (1 = positive, 0 = negative)	− .06	− .23 to .12	− .14^†^	− .28 to .004
R^2	.16		.33	
Distal factors				
Serum ferritin (µg/L)^1^			.46**	.37 to .55
Age (years)	− .06	− .17 to .04	− .05	− .13 to .03
Urinary cotinine (ng/mL)	.02	− .07 to .11	.19**	.13 to .26
Socioeconomic index	.20**	.09 to .31	.10*	.02 to .18
Recent pregnancy (12 months) (1 = yes, 0 = no)	− .34**	− .55 to − .13	− .15	− .34 to .04
Waist circumference > cut-off (1 = yes, 0 = no)	.30**	.13 to .48	.08	− .07 to .23
Food insecurity (1 = yes, 0 = no)	.08	** − **.13 to .29	.04	** − **.12 to .21
Country food rich in iron (frequency index)^3^	.01	** − **.09 to .10	.01	** − **.07 to .09
Hot beverages (frequency index)^3^	** − **.01	** − **.09 to .08	** − **.03	** − **.11 to .05
Alcohol consumption, drinks/day (1 = ≥ 1, 0 = < 1)	.38**	.19 to .57	.10	** − **.04 to .23
R^2	.15		.32	

Interaction term was tested for blood mercury and blood selenium but was not significant for both outcomes

^†^*p* < 0.1; *p < 0.05; **p < 0.01

^1^Log-transformed

^2^Sum of EPA, DHA, and DPA

^3^Index of frequency of consumption based on FFQ response (score from 0 to 7)

**Table 3:** Multiple logistic regression analyses of iron deficiency without anemia (IDWA) and iron deficiency anemia (IDA) among women of childbearing age (16 to 49 years old), Nunavik, 2017

	IDWA (*n* = 602)		IDA (*n* = 602)	
	***OR***	**95% *CI***	***OR***	**95% *CI***
Proximal factors				
Age (years)	0.92	0.70 to 1.19	1.13	0.81 to 1.58
Serum vitamin D (nmol/L)	1.21	0.87 to 1.69	0.57**	0.38 to 0.84
Blood mercury (nmol/L)^1^	0.90	0.65 to 1.23	0.86	0.60 to 1.23
Blood selenium (µmol/L)	0.73^†^	0.52 to 1.01	0.63^†^	0.39 to 1.04
hs-CRP, (1 = ≥ 3 mg/L, 0 = < 3 mg/L)	0.33**	0.16 to 0.67	1.50	0.80 to 2.83
RBC n-3 LC-PUFA (% of total fatty acids)^2^	0.66*	0.45 to 0.98	0.75	0.50 to 1.12
*H. pylori* serology (1 = positive, 0 = negative)	0.81	0.47 to 1.41	2.18*	1.13 to 4.23
Distal factors				
Age (years)	1.12	0.83 to 1.51	1.28	0.91 to 1.79
Socioeconomic index	0.76^†^	0.54 to 1.05	0.53*	0.32 to 0.87
Recent pregnancy (12 months), (1 = yes, 0 = no)	1.81^†^	0.98 to 3.34	2.27*	1.19 to 4.31
Waist circumference > cut-off, (1 = yes, 0 = no)	0.49*	0.28 to 0.85	0.78	0.45 to 1.35
Food insecurity, (1 = yes, 0 = no)	1.16	0.58 to 2.31	0.83	0.38 to 1.82
Country food rich in iron (frequency index)^3^	0.97	0.72 to 1.30	0.86	0.62 to 1.18
Hot beverages (frequency index)^3^	0.98	0.74 to 1.30	1.08	0.82 to 1.41
Alcohol consumption, drinks/day (1 = ≥ 1, 0 = < 1)	0.65	0.37 to 1.14	0.47*	0.25 to 0.88

Interaction term was tested for blood mercury and blood selenium but was not significative for both outcomes

^†^*p* < 0.1; *p < 0.05; **p < 0.01

^1^Log-transformed

^2^Sum of EPA, DHA, and DPA

^3^Index of frequency of consumption based on FFQ response (score from 0 to 7)

#### Women of childbearing age

In models focusing on proximal factors, serum vitamin D was associated with higher SF concentrations (Table 2) and with lower prevalence of IDA (Table 3). Blood Se was positively associated with SF concentrations, and marginally associated with lower IDWA and IDA prevalence. RBC n-3 LC-PUFAs were associated with higher SF levels and a lower prevalence of IDWA. Inflammatory status (hs-CRP ≥ 3 mg/L) was associated with higher SF and lower prevalence of IDWA but was associated with lower Hb levels. *H. pylori* serology was marginally associated with lower Hb and was positively associated with IDA. Urinary cotinine was positively associated with Hb.

In the models pertaining to distal factors, obesity was associated with higher concentrations of SF, and negatively associated with prevalence of IDWA. Pregnancy within the last 12 months was associated with lower SF concentrations and marginally associated with higher prevalence of IDWA and IDA. SES, based on education and income, was positively associated with SF and Hb, and negatively associated with IDA and marginally with IDWA. Finally, alcohol consumption was associated with higher SF concentrations and a decreased prevalence of IDA.

#### Younger men

Proximal factor models revealed that age was negatively associated with Hb but positively associated with SF (Table 4). Blood Se was positively associated with SF concentrations. A statistically significant interaction was noted between blood Hg and blood Se concentrations on SF. Indeed, the association between blood Se and SF was stronger when blood Hg concentrations were lower (− 1 SD; *β* = 0.88) and was weaker when blood Hg concentrations were higher (+ 1 SD; *β* = 0.34). The interaction plot is shown in Figure S1 of Supplementary Materials. *H. pylori* serology was negatively associated with SF, while inflammatory status was marginally associated with higher SF.

**Table 4:** Multiple linear regression analyses of serum ferritin and blood hemoglobin concentrations among younger men (16 to 49 years old), Nunavik, 2017

	Ferritin (µg/L)^1^ (*n* = 306)		Hemoglobin (g/L) (*n* = 306)	
	***Std β***	**95% *CI***	***Std β***	**95% *CI***
Proximal factors				
Serum ferritin (µg/L)^1^			.46**	.29 to .63
Age (years)	.18**	.06 to .30	− .13*	− .25 to − .02
Urinary cotinine (ng/mL)	− .05	− .17 to .07	.001	− .10 to .10
Serum vitamin D (nmol/L)	.03	− .12 to .18	− .01	− .13 to .11
Blood mercury (nmol/L)^1^	− .24^†^	− .50 to .02	.01	− .15 to .17
Blood selenium (µmol/L)	.61**	.23 to .99	.05	− .11 to .21
Blood mercury^1^ and selenium interaction	− .27*	− .50 to − .03		
hs-CRP, (1 = ≥ 3 mg/L, 0 = < 3 mg/L)	.30^t^	− .02 to .62	− .14	− .46 to .17
RBC n-3 LC-PUFA (% of total fatty acids)^2^	.07	− .10 to .23	− .01	− .15 to .14
*H. pylori* serology (1 = positive, 0 = negative)	− .29*	− .57 to − .01	.11	− .16 to .39
R^2	.16		.23	
Distal factors				
Serum ferritin (µg/L)^1^			.43**	.25 to .61
Age (years)	.14^†^	− .01 to .29	− .12*	− .24 to − .01
Urinary cotinine (ng/mL)	− .04	− .17 to .08	.01	− .09 to .12
Socioeconomic index	.11^t^	− .01 to .23	.09	− .02 to .20
Waist circumference > cut-off (1 = yes, 0 = no)	.38*	.05 to .72	.21	− .11 to .53
Food insecurity (1 = yes, 0 = no)	− .24	− .56 to .08	− .09	− .39 to .20
Country food rich in iron (frequency index)^3^	− .02	− .15 to .11	.10^†^	− .01 to .20
Hot beverages (frequency index)^3^	− .01	− .13 to .12	− .12^†^	− .24 to .01
Alcohol consumption, drinks/day (1 = ≥ 1, 0 = < 1)	.26*	.01 to .51	− .03	− .25 to .19
R^2	.14		.26	

^†^*p* < 0.1, *p < 0.05, **p < 0.01

^1^Log-transformed

^2^Sum of EPA, DHA, and DPA

^3^Index of frequency of consumption based on FFQ response (score from 0 to 7)

As for distal factors, obesity was positively associated with SF and SES marginally associated with higher SF. Although only marginally significant, country food consumption was positively associated with Hb, while hot beverage consumption was negatively associated with Hb. Finally, alcohol was positively associated with SF.

#### Older adults

In proximal factor models, age was negatively associated with Hb among older women (Table 5) and men (Table 6) but positively associated with SF in older women only. Inflammatory status was negatively associated with Hb among men and positively associated with SF among women. Urinary cotinine was associated with higher Hb concentrations, but only among women. *H. pylori* was positively associated with Hb in men.

**Table 5:** Multiple linear regression analyses of serum ferritin and blood hemoglobin concentrations among older women (50 years and older), Nunavik, 2017

	Ferritin (µg/L)^1^ (*n* = 229)		Hemoglobin (g/L) (*n* = 229)	
	***Std β***	**95% *CI***	***Std β***	**95% *CI***
Proximal factors				
Blood ferritin (µg/L)^1^			.22^**^	.10 to .33
Age (years)	.16^*^	.02 to .31	− .30^**^	− .41 to − .19
Urinary cotinine (ng/mL)	− .13	− .28 to .02	.12^*^	.01 to .22
Serum vitamin D (nmol/L)	− .05	− .19 to .09	− .02	− .15 to .11
Blood mercury (nmol/L)^1^	− .02	− .21 to .17	.12	− .03 to .27
Blood selenium (µmol/L)	.09	− .05 to .23	.01	− .10 to .13
hs-CRP, (1 = ≥ 3 mg/L, 0 = < 3 mg/L)	.29^*^	.02 to .56	.01	− .22 to .23
RBC n-3 LC-PUFA (% of total fatty acids)^2^	.09	− .11 to .28	.03	− .13 to .19
*H. pylori* serology (1 = positive, 0 = negative)	.19	− .06 to .44	.03	− .18 to .24
R^2	.10		.21	
Distal factors				
Blood ferritin (µg/L)^1^			.20**	.08 to .31
Age (years)	.16*	.03 to .29	− .25**	− .35 to − .14
Urinary cotinine (ng/mL)	− .12^†^	− .25 to .02	.12*	.01 to .23
Socioeconomic index	.07	− .07 to .21	.09^†^	− .01 to .19
Waist circumference > cut-off (1 = yes, 0 = no)	.33*	.05 to .61	.13	− .09 to .35
Food insecurity (1 = yes, 0 = no)	− .36*	− .63 to − .08	− .02	− .24 to .20
Country food rich in iron (frequency index)^3^	.07	− .06 to .20	.08	− .02 to .18
Hot beverages (frequency index)^3^	.04	− .11 to .19	− .09	− .20 to .02
Alcohol consumption, drinks/day (1 = ≥ 1, 0 = < 1)	.07	− .26 to .41	.16	− .11 to .42
Antacid medication, (1 = yes, 0 = no)	− .46^†^	− .93 to .01	.16	− .14 to .46
R^2	.16		.23	

Interaction term was tested for blood mercury and blood selenium but was not significant for both outcomes

^†^*p* < 0.1; *p < 0.05; **p < 0.01

^1^Log-transformed

^2^Sum of EPA, DHA, and DPA

^3^Index of frequency of consumption based on FFQ response (score from 0 to 7)

**Table 6:** Multiple linear analyses of serum ferritin and blood hemoglobin concentrations among older men (50 years and older), Nunavik, 2017

	Ferritin (µg/L)^1^ (*n* = 130)		Hemoglobin (g/L) (*n* = 130)	
	***Std β***	**95% *CI***	***Std β***	**95% *CI***
Proximal factors				
Blood ferritin (µg/L)^1^			.42**	.23 to .61
Age (years)	− .10	− .38 to .17	− .22*	− .45 to − .01
Urinary cotinine (ng/mL)	− .22^†^	− .44 to .01	.04	− .13 to .22
Serum vitamin D (nmol/L)	.11	− .19 to .41	− .02	− .27 to .22
Blood mercury (nmol/L)^1^	.09	− .24 to .42	.17	− .12 to .45
Blood selenium (µmol/L)	.08	− .20 to .36	− .03	− .38 to .31
hs-CRP, (1 = ≥ 3 mg/L, 0 = < 3 mg/L)	.38	− .13 to .89	− .63**	− .99 to − .26
RBC n-3 LC-PUFA (% of total fatty acids)^2^	− .09	− .37 to .19	− .06	− .32 to .21
*H. pylori* serology (1 = positive, 0 = negative)	.05	− .41 to .52	.42*	.07 to .78
R^2	.09 (n.s.)		.36	
Distal factors				
Blood ferritin (µg/L)^1^			.35**	.14 to .56
Age (years)	.03	− .22 to .29	− .30**	− .51 to − .09
Urinary cotinine (ng/mL)	− .08	− .30 to .15	.18	− .04 to .39
Socioeconomic index	− .03	− .29 to .22	.04	− .20 to .28
Waist circumference > cut-off (1 = yes, 0 = no)	.24	− .23 to .71	.32	− .12 to .77
Food insecurity (1 = yes, 0 = no)	− .64**	− 1.16 to − .12	− .07	− .52 to .39
Country food rich in iron (frequency index)^3^	− .001	− .22 to .22	− .07	− .25 to .12
Hot beverages (frequency index)^3^	.10	− .12 to .32	.01	− .20 to .21
Alcohol consumption, drinks/day (1 = ≥ 1, 0 = < 1)	.40	− .09 to .88	.11	− .37 to .58
Antacid medication (1 = yes, 0 = no)	− .39	− .99 to .11	− .17	− .71 to .37
R^2	.14 (n.s.)		.30	

Interaction term was tested for blood mercury and blood selenium but was not significant for both outcomes

^†^*p* < 0.1; *p < 0.05; **p < 0.01

^1^Log-transformed

^2^Sum of EPA, DHA, and DPA

^3^Index of frequency of consumption based on FFQ response (score from 0 to 7)

In models focusing on distal factors, food insecurity was negatively associated with SF among older women and men. Among older women, obesity was associated with higher SF concentrations, and taking an antacid medication was marginally associated with lower SF concentrations.

## Discussion

### Iron status and anemia among Inuit adults in Nunavik

High prevalence of ID and anemia have been documented in the Circumpolar Arctic for decades, even though the traditional Inuit diet includes a variety of animal-based country foods that are rich in heme iron and multiple essential nutrients. Compared to the 2004 survey (Plante et al., 2011), anemia among Nunavik women was about 50% less prevalent in 2017, no matter the age group; the prevalence of ID also decreased by approximately 30% between 2004 and 2017, but the reduction was mainly confined to women aged 18 to 29 (Table 7). While caution should be exerted when comparing data from two different surveys, both *Qanuippitaa?* 2004 and *Qanuilirpitaa?* 2017 are population-based surveys that were conducted during the same period of the year (from late summer to early fall), whose design and weighting process were elaborated by the Institut national de santé publique du Québec (Hamel et al., 2020; Rochette & Blanchet, 2007). Laboratory methods used in both surveys to assess anemia and iron status were similar and involved extensive quality control procedures. Therefore, we are confident that the markedly lower prevalence of anemia and ID observed in 2017 truly reflect an improvement of these conditions compared to 2004. The prevalence of ID and anemia documented in Nunavik in 2017 were similar to those in the other regions of the Inuit Nunangat in 2007–2008, both globally and for the different sex and age groups (Jamieson et al., 2012, 2013), but still much higher than in the general Canadian population (Cooper et al., 2012).

**Table 7:** Comparison of the prevalence of anemia and iron deficiency in women 18 years and older in the *Qanuippitaa?* 2004 and the *Qanuilirpitaa?* 2017 surveys

	***Qanuippitaa?* 2004**^**1**^		***Qanuilirpitaa?* 2017**	
	***Prevalence (%)***	***95% CI***	***Prevalence (%)***	***95% CI***
Anemia				
18–29 y	41.7	34.8 to 49.0	19.8**	15.3 to 25.2
30–49 y	39.5	33.7 to 45.7	18.3**	13.6 to 24.2
50–74 y	60.8	51.9 to 69.1	28.6**	22.3 to 35.9
Total	44.8	41.0 to 48.6	21.6**	18.5 to 25.0
Iron deficiency				
18–29 y	47.1	40.7 to 53.6	30.6**	25.5 to 36.2
30–49 y	36.0	30.3 to 42.2	30.4	24.6 to 37.0
50–74 y	9.8*	5.7 to 16.4	7.2*	4.0 to 12.5
Total	34.4	31.0 to 38.0	24.2**	21.1 to 27.7

^1^Data source: *Qanuippitaa?* 2004 (unpublished results)

^*^Coefficient of variation greater than 25%. Proportion is shown for information only

^**^Statistically significant difference between prevalence in 2004 and 2017 based on non-overlapping 95% confidence intervals

Women of childbearing age exhibited the highest prevalence of ID (33%), IDWA (23%), and IDA (14%). Indeed, SF concentrations were significantly lower among women of childbearing age, and age was positively associated with SF among older women. After menopause, iron loss and requirements decrease as a result of cessation of menstruation, resulting in a rise of iron stores (Scientific Advisory Committee on Nutrition, 2010).

Men and women aged 50 and older had a high prevalence of anemia (31%), most cases being unrelated to ID. Similarly, data from the Inuit Health Survey (IHS) conducted in 2007–2008 showed that the prevalence of anemia among Inuit men (30%) and women (25%) over 50 years of age in the Inuit Nunangat, excluding Nunavik, was the highest, despite having the lowest prevalence of iron depletion (Jamieson et al., 2012, 2013). Higher prevalence of anemia among older adults was also reported in various Inuit (Milman et al., 2001; Plante et al., 2011) and non-Inuit populations (Cooper et al., 2012; McLean et al., 2009). Anemia in older adults is often associated with increased disability, mortality, and hospitalization and should not be perceived as simply a normal part of ageing.

### Protective and risk factors in Nunavik

#### Proximal factors—country food nutrients

Vitamin D was associated with higher SF and lower IDA prevalence among women of childbearing age. Similarly, data from the Korean National Health and Nutrition Examination Survey in 2012 revealed that vitamin D concentrations were associated with increased SF and Hb. Vitamin D suppresses hepcidin, leading to increased ferroportin expression, the chief protein involved in iron exportation from enterocytes or storage sites, resulting in increased iron in the systemic circulation (Zughaier et al., 2014).

Blood Se was positively associated with SF among younger men and women. In Nunavik, blood selenium is mainly present in the form of selenoneine, an organoselenium compound that accumulates in RBCs (Achouba et al., 2019) and exhibits antioxidant properties, which could protect against oxidation and prolong RBC survival (Yamashita et al., 2010), and in turn reduces iron requirements for erythropoiesis. Moreover, binding of selenoneine to heme proteins could limit toxicity of methylmercury (MeHg), a contaminant present in some country food, as MeHg is known to bind to and interfere with Hb in RBCs (Yamashita & Yamashita, 2010). Interestingly, the positive association between blood Se and SF in young men was stronger at lower blood Hg levels, although this interaction was not consistently observed among other subgroups, which presented higher blood Se and Hg concentrations. Additional studies are needed to further elucidate these findings and the protective role of selenoneine on ID and anemia as well as Hg-Se interactions in Nunavimmiut RBC.

RBC n-3 LC-PUFA were positively associated with SF concentration, and negatively associated with the prevalence of IDWA, but not IDA among women of childbearing age. Jamieson et al. (2013) also reported that EPA and DPA were associated with a reduced risk of ID in Inuit women in IHS. In Nunavik, the main source of n-3 LC-PUFA is the consumption of marine mammals and fish, which are also rich in iron (Lemire et al., 2015). n-3 LC-PUFA have also been shown to downregulate inflammatory processes and subsequently improve iron homeostasis (Bersamin et al., 2008).

#### Proximal factors—inflammation and *H. pylori*

Inflammatory status (hs-CRP ≥ 3 mg/L) was associated with higher SF among all Nunavimmiut except older men, and with lower Hb among older men and women of childbearing age. These results were expected as SF is an acute-phase reactant that increases during inflammation. Hb is expected to decrease as mobilization of iron for erythropoiesis is diminished in the presence of inflammation, even in the presence of adequate iron stores (Nemeth & Ganz, 2006).

*H. pylori*-positive serology, reflecting a past or active infection, was most prevalent and negatively associated with SF among younger men, while it was positively associated with IDA among women of childbearing age. Similarly, *H. pylori*-positive serology was also associated with lower SF among Inuit from the IHS (Jamieson et al., 2012, 2013). *H. pylori* colonizes the gastric mucosa, and while *H. pylori* infection is often asymptomatic, it can lead to ID or anemia due to gastrointestinal blood loss, the disturbance of normal iron metabolism processes, or bacterial sequestration of free iron (Barabino, 2002). *H. pylori-*positive serology was unexpectedly positively associated with Hb concentrations among older men. This was also reported in two studies in Haiti and Zanzibar (Farag et al., 2007; Shak et al., 2011); it was speculated that higher bacterial loads could increase the secretion of gastric acid, which promotes iron absorption.

#### Distal factors—SES and food security

A higher SES, based on income and education, was associated with higher SF and/or Hb concentrations among all Nunavimmiut except older men. These results are consistent with those of 2004 in Nunavik (Plante et al., 2007), and in other Inuit (Jamieson et al., 2012, 2013, 2016) and non-Inuit populations (Cooper et al., 2012). Lower SES can limit the capacity to buy food and access nutritious food. Indeed, Huet et al. (2012) found that the presence of at least one indicator of socioeconomic disadvantage was associated with food insecurity and low-quality diet. Moreover, in our study, food insecurity itself was associated with lower SF concentrations among older adults. Similarly, in a study conducted among school-aged children in Nunavik, food insecurity was associated with a higher prevalence of ID and anemia (Pirkle et al., 2014). The prevalence of food insecurity in Nunavik is very high (Table 1), leading to an inadequate access to sufficient nutritious (iron-rich) and culturally preferred food (Arriagada, 2017).

#### Distal factors—diet and lifestyle

Obesity was positively associated with SF among women of all ages and among younger men. These results agree with those of Jamieson et al. (2012, 2013) who reported a negative association between adiposity and ID among Inuit adults. In contrast, other studies among non-Inuit populations have previously reported that obesity could contribute to ID and anemia by increasing hepcidin expression and disturbing systemic iron homeostasis (Nead et al., 2004; Scheer & Guthrie, 1981). In our study, younger men and women who were obese had a higher prevalence of inflammation compared to those who were not obese (data not shown). Similar to hs-CRP, SF is an acute-phase reactant and the positive association between obesity and SF observed in the present study could therefore partially be due to inflammation.

Excessive alcohol intake is a recognized risk factor for anemia, especially megaloblastic anemia (Ballard, 1997). In the present study, this form of anemia was not observed (Lavoie et al., 2020). Alcohol consumption was rather associated with an increase in SF among younger men and women, and a decreased risk of IDA in women of childbearing age. Similarly, Ioannou et al. (2004), who analyzed data from the NHANES III, found that alcohol consumption among adults aged 16 and older, regardless of quantity, reduced the risk of IDA by 40%. Alcohol is a known iron absorption enhancer, increasing gastric acid secretion and, hence, iron solubilization. Alcohol has also been shown to reduce hepcidin concentrations, leading to increased expression of iron transport proteins (DMT1 and ferroportin), and further increased SF (Harrison-Findik, 2007).

In the present survey, among younger men, the consumption of country food rich in iron was marginally associated with higher Hb, while tea and coffee consumption was marginally associated with lower Hb. Among Inuit men from the IHS 2007–2008, consumption of tea was marginally associated with decreased SF; however, the association between tea consumption and Hb was not reported (Jamieson et al., 2012). Both tea and coffee contain tannins and polyphenols, which both bind to free iron in the intestinal lumen, thereby limiting its absorption (Hurrell et al., 1999), which could then decrease Hb.

### Perspectives on improving iron status and decreasing the prevalence of anemia in Nunavik

Different initiatives implemented over the years may have contributed to improving iron status and decreasing the prevalence of anemia in Nunavik. Inuit-led initiatives to improve food security include food banks, soup kitchens, community harvesting initiatives, nutrition education initiatives, and school breakfast programs. These food security initiatives are intended to alleviate hunger and provide short-term and direct relief to individuals and families. Others teach food skills, such as nutrition, cooking, country food harvesting, and gardening skills. Regional hunter support programs encourage harvesting as a way of life, and community freezers provide country food to Inuit households (ITK, 2021). The *Ilagiilluta* program, which is being gradually implemented in Nunavik, uses a community development approach to promote physical and mental well-being of families by offering supportive services tailored to their needs. The program targets all pregnant women and families with young children. Nutrition counselling, support, education, referral, and counselling on lifestyle issues are among the services provided by the program. In all Nunavik communities, healthy food coupons are provided during pregnancy through funding from the Canadian Prenatal Nutrition Program (CPNP, 2022; PIWC, 2021). More in-depth analyses are required to further pinpoint the factors responsible for the seemingly improved iron status and hemoglobin levels among women since the 2004 survey.

Although several preventive and risk factors of ID and anemia have been identified in the present study, SES and food security stand out as the most important factors across different subgroups. Access to country food and other nutritious foods as well as increasing SES should continue to be prioritized to fight against ID and anemia among Inuit of all ages.

### Limitations

The study is based on a cross-sectional design, limiting inferences about the direction of associations and causality. The low global response rate noted in the present study (37%) increases the risk of a selection bias, which may not have been efficiently corrected by statistical weighting. Nunavimmiut who participated in the survey might differ from the adult Nunavik population with regard to the outcomes (ID and anemia) and proximal/distal determinant profile. However, the population sample recruited for the *Qanuilirpitaa?* 2017 survey was diverse with regard to various factors including socioeconomic status, food security status, dietary habits, and Inuit culture adherence (data available in the following thematic reports: Allaire et al., 2021; Furgal et al., 2021; Muckle et al., 2020; Riva et al., 2020). Discussions of survey results during co-interpretation sessions were supportive of the population sample adequately reflecting the diversity of sociodemographic and socio-cultural characteristics as well as lifestyle habits of Nunavimmiut. The FFQ questionnaire did not take portions into account, limiting the interpretation of associations observed with dietary variables. Finally, although regressions were separated into two separate models to consider the effect of more distal and more proximal variables, there are probably many more layers of variables in the causal pathway to ID and anemia. In the future, the use of structural equation modelling could help better identify various moderation effects in the causal chain leading to ID and anemia.

## Conclusion

Even though there was an apparent improvement with regard to iron deficiency and anemia in Nunavik women between 2004 and 2017, both conditions remain highly prevalent as in other Inuit populations. In particular, the elevated prevalence of anemia among women of childbearing age (20%) and older Nunavimmiut (31%) warrants further investigation, as it still represents a moderate public health problem according to WHO criteria (Guralnik et al., 2004; Stauder & Thein, 2014; WHO, 2017). Programs tackling food insecurity and poverty should be maintained and expanded in Nunavik.

## Contributions to knowledge

What does this study add to existing knowledge?

- Anemia remains common in Nunavik, especially among women of childbearing age and older adults, constituting a moderate public health problem according to World Health Organization criteria.
- Several nutrients present in country foods seem to protect Nunavimmiut women of childbearing age against iron deficiency.
- Low socioeconomic status and food insecurity are the most important risk factors of anemia and iron deficiency across the different population subgroups.

What are the key implications for public health interventions, practice, or policy?

- Findings can be used to push for more effective policy measures to reduce poverty and improve access to country foods and nutrient-rich market foods.
- Strengthening the Nunavik food system in partnership with Inuit organizations will contribute to reducing iron deficiency and anemia as well as other important health issues in the region.

### Supplementary Information

Below is the link to the electronic supplementary material.

Supplementary file1 (DOCX 79.8 kb)

## Data Availability

The survey data are owned by Inuit and can be accessed through a request made to *Qanuilirpitaa?* 2017 DMC (nunavikhealthsurvey@ssss.gouv.qc.ca).
