# Supplementary material for: Determinants of iron deficiency and anemia among Nunavimmiut: results from the Qanuilirpitaa? 2017 Nunavik Health Survey
Source: Can J Public Health. 2023 May 10;115(Suppl 1):152–67. doi: 10.17269/s41997-023-00775-4 (PMC10831004; doi:10.17269/s41997-023-00775-4)
Supplement: Supplementary file 1 — Supplementary file1 (DOCX 79.8 kb) [file 41997_2023_775_MOESM1_ESM.docx]

Determinants of Iron Deficiency and Anemia among Nunavimmiut:
Results from the *Qanuilirpitaa?* 2017 Nunavik Health Survey

**Audrey Lavoie, MSc**

Département de médecine sociale et préventive, Université Laval, Quebec City, QC, Canada

Email: [audrey.lavoie.20@ulaval.ca](mailto:audrey.lavoie.20@ulaval.ca)

**Mélanie Lemire, PhD, Associate Professor**

Département de médecine sociale et préventive and Institut de biologie intégrative et des systèmes, Université Laval

Axe santé des populations et pratiques optimales en santé – Centre de recherche du CHU de Québec – Université Laval

Email: [melanie.lemire@crchudequebec.ulaval.ca](mailto:melanie.lemire@crchudequebec.ulaval.ca); ORCID (16-digit): [0000-0003-3334-1349](https://orcid.org/0000-0003-3334-1349)

**Benoit Lévesque, MD, MSc**

Institut national de santé publique du Québec, Quebec City, QC, Canada

Département de médecine sociale et préventive, Université Laval

Email: [benoit.levesque2402@](mailto:benoit.levesque2402@)gmail.com; ORCID (16-digit): 0000-0002-8125-6635

**Pierre Ayotte, PhD, Professor, corresponding author**

Département de médecine sociale et préventive, Université Laval

Axe santé des populations et pratiques optimales en santé – Centre de recherche du CHU de Québec – Université Laval

Institut national de santé publique du Québec

Email: [pierre.ayotte@inspq.qc.ca](mailto:pierre.ayotte@inspq.qc.ca); ORCID (16-digit): 0000-0003-1970-7252

Phone number: 418-650-5115 #4654

**Table of contents**

[**Description of proximal and distal factors** 2](#_Toc133249377)

[**Construction of the socio-economic index** 5](#_Toc133249378)

[**Table S1.** Analytical performance of method M-592 for the determination of mercury (Hg), lead (Pb) and selenium (Se) in whole blood 6](#_Toc133249379)

[**Table S2.** Percentage of missing data for variables in statistical models 7](#_Toc133249380)

[**Figure S1.** Standardized interaction plot of the relationship between blood selenium and serum ferritin levels according to blood mercury levels among Inuit men aged 16 to 49 years, Nunavik, 2017 8](#_Toc133249381)

# **Description of proximal and distal factors**

Proximal factors

Inflammation is the second most common cause of anemia, after ID (Jamieson, 2012). In the presence of inflammation, hepcidin – a peptide hormone released by the liver – is elevated and disturbs iron absorption, iron recycling and iron storage mobilization, leading to reduced circulating iron, despite adequate stored iron (Nemeth & Ganz, 2006). Obesity has been associated with a state of low-grade inflammation, which contributes to the development of ID and anemia (Aigner et al., 2014). Clinical inflammation, associated with infections and chronic diseases, is a well-known risk factor of anemia (Christofides et al., 2005). *Helicobacter pylori* (*H. pylori*) is a gastrointestinal infection that is highly prevalent in the Arctic (Goodman et al., 2008) and known to be associated with ID and anemia through inflammation but other mechanisms as well. First, *H. pylori* causes gastric hypoacidity, which reduces iron absorption (Barabino, 2002). Antacid medication, which is often prescribed to treat *H. pylori*, can also contribute to this hypoacidity (Sarzynski et al., 2011). Second, the bacteria compete for iron, limiting available iron for bodily functions. Third, iron and Hb can be loss by gastrointestinal bleeding (Barabino, 2002).

Other nutrient deficiencies, such as vitamin B12, folate, vitamin D and selenium (Se) deficiencies are also potential causes of ID and anemia. Indeed, vitamin B12 and folate deficiencies both alter the DNA synthesis process of red blood cells (RBCs), creating large RBC precursors, leading to megaloblastic anemia (Fishman et al., 2000). Moreover, vitamin D has anti-inflammatory properties; and therefore, could protect against anemia of chronic inflammation (Sim et al., 2010). Vitamin D also has a direct role in erythropoiesis, stimulating erythroid precursor proliferation (Alon et al., 2002). Se is well known for its antioxidant properties, and Se deficiency has been identified as a risk factor of anemia and ID in various populations (Larvie et al., 2019; Semba et al., 2006; Van Nhien et al., 2009).

The Inuit traditional diet is made up of marine foods such as marine mammals and fish, both being exceptionally rich in omega-3 long chain polyunsaturated fatty acids (n-3 LC-PUFA) (Lemire et al., 2015). RBC n-3 LC-PUFA have been positively associated with SF in both, non-Inuit (Diaz-Castro et al., 2015) and Inuit studies (Jamieson et al., 2013b, 2016). It was also suggested by Jamieson et al. (2016) that an increase in RBC eicosapentaenoic acid (EPA) – one of the n-3 LC-PUFAs – could increase RBC oxidative stress and lead to accelerated RBC turnover (Miret et al., 2003), therefore reducing blood Hb concentrations.

Elevated exposure to methylmercury (MeHg), which greatly bioaccumulate in few marine mammals and predatory fish species, may contribute to anemia as MeHg competes with iron to form a strong bond with Hb, which leads to inefficient Hb compounds (Rice et al., 2014). Furthermore, exposure to high levels of lead (Pb) can disturb heme synthesis and accelerate RBC destruction (Shah et al., 2010).

Distal factors

The Inuit traditional diet is rich in animal-based foods, which are excellent sources of heme iron and several nutrients mentioned above (Kuhnlein et al., 2004; Lemire et al., 2015). Indeed, country food consumption has been positively associated with SF ad Hb concentrations among Inuit living in the Canadian Arctic (Jamieson et al., 2012, 2016). Some food components, such as tannins and polyphenols in hot beverages and phytates in cereal products are iron inhibitors, reducing iron absorption. On the other hand, vitamin C in fruits, vegetables and juices is known to increase iron absorption (Hurrell & Egli, 2010). Alcohol intake has also been shown to increase iron absorption by decreasing hepcidin expression (Harrison-Findik et al., 2006).

Women of childbearing age are particularly at risk for ID and anemia due to iron loss during menstruation and increased iron requirements during and after pregnancy. During pregnancy, iron needs are greatly increased and hemodilution reduces Hb concentrations (Sharma & Shankar, 2010). Additionally, if breastfeeding, most of the mother’s iron stores will be used for breast milk, regardless of the mother’s current iron stores (Lakew et al., 2015). Recent pregnancy has previously been associated with higher prevalence of ID and IDA among women in Nunavik (Plante et al., 2007).

Several social determinants of health have also been linked to ID and anemia in both non-Inuit (Braveman & Gottlieb, 2014) and Inuit populations (Garner et al., 2010). In 2004, a lower socioeconomic status (SES) was associated with a higher prevalence of ID and anemia among women in Nunavik (Plante et al., 2007). A low SES has also been associated with an increased risk of food insecurity, a widely present issue in Nunavik (Jamieson et al., 2012). In turn, food insecurity can reduce food intake, yielding inadequate intake of various nutrients required for erythropoiesis, and subsequently to ID and anemia (Pirkle et al., 2014a).

References

Aigner, E., Feldman, A., & Datz, C. (2014). Obesity as an emerging risk factor for iron deficiency. *Nutrients Journal*, 6(9), 3587–3600. https:// doi. org/ 10. 3390/ nu609 3587

Alon, D. B., Chaimovitz, C., Dvilansky, A., Lugassy, G., Douvdevani, A., Shany, S., & Nathan, I. (2002). Novel role of 1,25(OH)2D3 in induction of erythroid progenitor cell proliferation. *Experimental Hematology*, 30(5), 403–409. https:// doi. org/ 10. 1016/ S0301-472X(02) 00789-0

Barabino, A. (2002). Helicobacter pylori-related iron deficiency anemia:A review. *Helicobacter*, 7(2), 71–75. https:// doi. org/ 10. 1046/j. 1083- 4389. 2002. 00073.x

Braveman, P., & Gottlieb, L. (2014). The social determinants of health: It’s time to consider the causes of the causes. *Public Health Reports*, 129(2), 19–31. https:// doi. org/ 10. 1177/ 00333 54914 1291s 206

Christofides, A., Schauer, C., & Zlotkin, S. H. (2005). Iron deficiency anemia among children: Addressing a global public health problem within a Canadian context. *Paediatrics & Child Health*, 10, 597–601. https:// doi. org/ 10. 1093/ pch/ 10. 10. 597

Garner, R., Carrière, G., Sanmartin, C. A., & Team, L. R. (2010). The health of first nations living off-reserve, Inuit, and Métis Adults in Canada: The impact of socio-economic status on inequalities in health. Statistics Canada.

Fishman, S. M., Christian, P., & West, K. P. (2000). The role of vitamins in the prevention and control of anaemia. *Public Health Nutrition*, 3(2), 125–150. https:// doi. org/ 10. 1017/ S1368 98000 00001 73

Goodman, K. J., Jacobson, K., & van Zanten, S. V. (2008). Helicobacter pylori infection in Canadian and related Arctic aboriginal populations. *Canadian Journal of Gastroenterology*, 22(3). https:// doi. org/ 10. 1155/ 2008/ 258610

Harrison-Findik, D. D., Schafer, D., Klein, E., Timchenko, N. A., Kulaksiz, H., Clemens, D., . . . Gollan, J. (2006). Alcohol metabolism- mediated oxidative stress down-regulates hepcidin transcription and leads to increased duodenal iron transporter expression. *Journal of Biological Chemistry*, 281(32), 22974–22982. doi:https:// doi. org/ 10. 1074/ jbc. M6020 98200

Jamieson, J. A. (2012). Correlates of iron status, hemoglobin and anemia in Inuit adults. (Master's thesis). McGill University (Canada), Montreal

Jamieson, J. A., Weiler, H. A., Kuhnlein, H. V., & Egeland, G. M. (2016). Prevalence of unexplained anaemia in Inuit men and Inuit post-menopausal women in Northern Labrador: International Polar Year Inuit Health Survey. *Canadian Journal of Public Health*, 107(1), e81-87. https:// doi. org/ 10. 17269/ cjph. 107. 5173

Hurrell, R., & Egli, I. (2010). Iron bioavailability and dietary reference values. *The American Journal of Clinical Nutrition*, 91(5), 1461S-1467S. https:// doi. org/ 10. 3945/ ajcn. 2010. 28674F

Kuhnlein, H. V., Receveur, O., Soueida, R., & Egeland, G. M. (2004). Arctic indigenous peoples experience the nutrition transition with changing dietary patterns and obesity. *Journal of Nutrition*, 134(6), 1447–1453. https:// doi. org/ 10. 1093/ jn/ 134.6. 1447

Lakew, Y., Biadgilign, S., & Haile, D. (2015). Anaemia prevalence and associated factors among lactating mothers in Ethiopia: Evidence from the 2005 and 2011 demographic and health surveys. *British Medical Journal Open*, 5(4), e006001–e006001. https:// doi. org/10. 1136/ bmjop en- 2014- 006001

Larvie, D. Y., Doherty, J. L., Donati, G. L., & Armah, S. M. (2019). Relationship between selenium and hematological markers in young adults with normal weight or overweight/obesity. *Antioxidants*, 8(10), 463. https:// doi. org/ 10. 3390/ antio x8100 463

Lemire, M., Kwan, M., Laouan-Sidi, A. E., Muckle, G., Pirkle, C., Ayotte, P., & Dewailly, E. (2015). Local country food sources of methylmercury, selenium and omega-3 fatty acids in Nunavik, Northern Quebec. *Science of the Total Environment*, 509–510, 248–259. https:// doi. org/ 10. 1016/j. scito tenv. 2014. 07. 102

Nemeth, E., & Ganz, T. (2006). Regulation of iron metabolism by hepcidin. Annual Review of Nutrition, 26, 323–342. https:// doi. org/ 10. 1146/ annur ev. nutr. 26. 061505. 111303

Pirkle, C. M., Lucas, M., Dallaire, R., Ayotte, P., Jacobson, J. L., Jacobson, S. W., & Muckle, G. (2014). Food insecurity and nutritional biomarkers in relation to stature in Inuit children from Nunavik. *Canadian Journal of Public Health*, 105(4), e233-238. https:// doi. org/ 10. 17269/ cjph. 105. 4520

Plante, C., Blanchet, C., & Turgeon O'Brien, H. (2007). *Qanuippitaa? 2004 Nunavik Inuit Health Survey: Iron deficiency and anemia among women in Nunavik*. Nunavik Regional Board of Health and Social Services & Institut national de santé publique du Québec. http:// nrbhss. ca/ en/ health- surve ys. Accessed January 31, 2023.

Sarzynski, E., Puttarajappa, C., Xie, Y., Grover, M., & Laird-Fick, H. (2011). Association between proton pump inhibitor use and anemia: A retrospective cohort study. *Digestive Diseases and Sciences*, 56(8), 2349–2353. https:// doi. org/ 10. 1007/s10620- 011- 1589-y

Semba, R. D., Ferrucci, L., Cappola, A. R., Ricks, M. O., Ray, A. L., Xue, Q. L., . . . Fried, L. P. (2006). Low serum selenium is associated with anemia among older women living in the community: The Women’s Health and Aging Studies I and II. *Biological Trace Element Research*, 112(2), 97–107. https:// doi. org/ 10. 1385/ bter: 112:2: 97

Shak, J. R., Sodikoff, J. B., Speckman, R. A., Rollin, F. G., Chery, M. P., Cole, C. R., & Suchdev, P. S. (2011). Anemia and Helicobacter pylori seroreactivity in a rural Haitian population. *American Journal of Tropical Medicine and Hygiene*, 85(5), 913–918. https:// doi. org/ 10. 4269/ ajtmh. 2011. 11- 0101

Sharma, J. B., & Shankar, M. (2010). Anemia in pregnancy. Journal *International Medical Sciences Academy*, 23(4), 253–260.

Sim, J. J., Lac, P. T., Liu, I. L. A., Meguerditchian, S. O., Kumar, V. A., Kujubu, D. A., & Rasgon, S. A. (2010). Vitamin D deficiency and anemia: A cross-sectional study. *Annals of Hematology*, 89(5), 447–452. https:// doi. org/ 10. 1007/ s00277- 009- 0850-3

Van Nhien, N., Yabutani, T., Khan, N. C., Khanh, L. N. B., Ninh, N. X., Chung, L. T. K., & Nakaya, Y. (2009). Association of low serum selenium with anemia among adolescent girls living in rural Vietnam. *Nutrition*, 25(1), 6–10. https:// doi. org/ 10. 1016/j. nut. 2008. 06. 032

# **Construction of the socio-economic index**

A socio-economic index was created for survey participants based on their answers to the questions on income and education in the table below. The score for income (0-1) was added to the score for education (0-1) and the sum was divided by 2 to yield the socio-economic index (0-1).

| **Income**  What is your best estimate of your total personal **income**, before taxes and other deductions, from all sources in the past 12 months?  0 = "Less than $20,000"  0.25 = "$20,000 to less than $40,000"  0.5 = "$40,000 to less than $60,000"  0.75 = "$60,000 to less than $80,000"  1 = "$80,000 or more" | **Education**  What is the **highest grade** you have completed?  0 = "Grade 1,2,3"  0.2 = "Grade 4,5,6"  0.4 = "Grade 7,8,9"  0.6 = "Grade 10,11(graduated) "  0.8 = "Some CEGEP/college" OR "Graduated from CEGEP/college"  1 = "Some University" OR "Graduated from University" |
| --- | --- |

| **Table S1.** Analytical performance of method M-592 for the determination of mercury (Hg), lead (Pb) and selenium (Se) in whole blood | | | |
| --- | --- | --- | --- |
| **Performance data** | Hg | Pb | Se |
| Limit of detection (nmol/L) | 3.0 | 70 | 300 |
| Repeatability (%)^1^ | 2.6 | 1.9 | 2.0 |
| Reproducibility (µg/L)^1^ | 6.0 | 3.8 | 4.0 |
| Accuracy (%)^2^ |  |  |  |
| PC-B-C1801 | -12 | 5.9 | NA |
| PC-B-C1802 | -10 | 0.6 | NA |
| PC-B-C1803 | -8.7 | -1.7 | NA |
| QM-B-Q1801 | -9.8 | -0.9 | -13 |
| QM-B-Q1802 | -7.1 | -1.8 | -16 |
| QM-B-Q1803 | -12 | 2.4 | -11 |

^1^ Repeatability and reproducibility at 6.4, 70 and 1800 nmol/L for Hg, Pb and Se, respectively.

^2^ Accuracy data from PCI and QMEQAS interlaboratory comparison programs (cycle #2018-01).

| **Table S2.** Percentage of missing data for variables in statistical models | |
| --- | --- |
| Variable | % data missing |
| Age (years) | 0 |
| Blood hemoglobin (g/L) | 0.08 |
| Serum ferritin (µg/L) | 0.08 |
| **Proximal factors** |  |
| Serum vitamin D (nmol/L) | 0.08 |
| Serum vitamin B12 (pmol/L) | 0.08 |
| RBC folate (nmol/L) | 0.08 |
| Blood total mercury (nmol/L) | 0.15 |
| Blood total lead (µmol/L) | 0.08 |
| Blood total selenium (µmol/L) | 0.08 |
| RBC n-3 LC-PUFA (% of total fatty acids) | 0.38 |
| Urinary cotinine (ng/mL) | 1.15 |
| hs-CRP | 0.08 |
| *H. pylori* serology | 1.15 |
| **Distal factors** |  |
| Waist circumference | 3.38 |
| Food insecurity | 7.15 |
| Income | 14.38 |
| Education | 2.92 |
| Country food rich in iron | 10.31 |
| Hot beverages, | 11.23 |
| Alcohol consumption | 6.23 |
| Antacid medication | 0 |
| Recent pregnancy (women 16 to 49 years of age) | 4.46 |

**Figure S1.** Standardized interaction plot of the relationship between blood selenium and serum ferritin levels according to blood mercury levels
among Inuit men aged 16 to 49 years, Nunavik, 2017


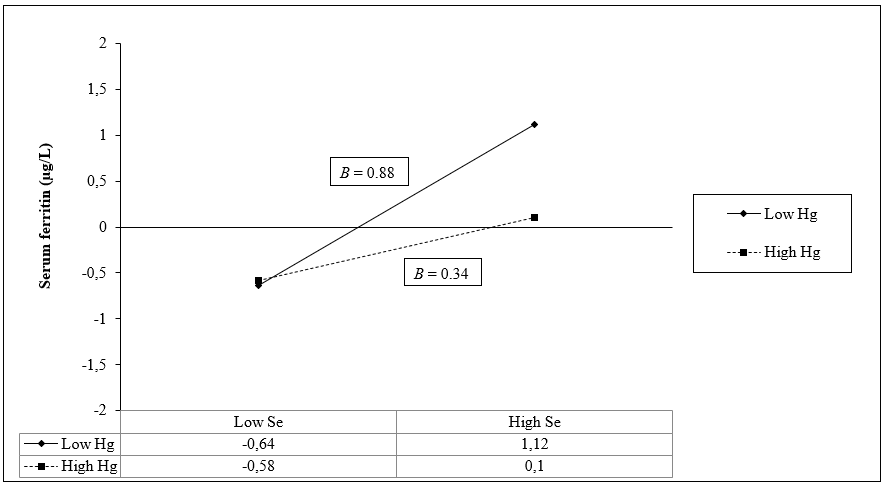


*Se = selenium, Hg = mercury; Low Hg = -1 SD, High Hg = +1 SD. Serum ferritin is log-transformed.*
